# Feeling like a ‘real’ scientist

**DOI:** 10.7554/eLife.77719

**Published:** 2022-02-17

**Authors:** Aalok Varma

**Affiliations:** 1 National Centre for Biological Sciences Bangalore India

**Keywords:** sparks of change, peer review, early career researchers, impostor syndrome

## Abstract

Peer reviewing helped a graduate student to finally gain a sense of belonging within the research community.

One of the best things about academia is being surrounded by smart people. Sometimes, it’s also one of the worst.

Early in my PhD, I would sit through seminars and just be in awe of senior students and professors. They would keep asking all these insightful questions while I just stared around, having understood only half the presentation (if that!) and wondering if I would ever be able to get to their level.

I had entered graduate school with brazen self-assurance, having always been an excellent student; in the few – or not so few – years since, that confidence has morphed into self-doubt, overthinking and impostor syndrome. My scientific output hasn’t grown as I hoped, projects have hit dead ends or got stuck. And then the pandemic hit, forcing me to start a completely new lockdown-friendly project to finish my PhD.

This is when I received a message from my graduate advisor: she was reviewing a paper for a journal and wanted me to help. I had a week to share my comments with her.

It was only the second time I had formally participated in reviewing a manuscript, and I felt the weight of the responsibility. Peer review (whether it happens before or after publication) is a cornerstone of the scientific process, a way for research to self-correct. I knew I needed to be a fair critic, but I also didn’t want to become one of those infamous, caustic ‘reviewers #2’.

I spent three days carefully going through the manuscript, covering the PDF with notes and markings. The paper was challenging, so I had to dive deep into textbooks, references and even a couple of YouTube videos to make sure I fully understood the complex methods that were used. After that first read, I went over the paper again and again, listing my comments and questions, often second-guessing myself: had I understood that part correctly? Had I missed something? Was I asking for something impossible – or worse, dumb? Despite my anxiety, this work was truly fulfilling. After all, constantly learning and being exposed to new concepts was why I had joined graduate school to begin with.

Having obsessed over the review enough, I finally managed to hit the ‘send’ button and share my report, expecting that this would be the end of it. I was aware that many researchers have reviewed papers on behalf of their advisors and have not been credited or acknowledged.

Yet a few days later, my supervisor got back in touch to share her own comments on the paper and to ask me to collate our two reviews. I reluctantly opened her document, fully expecting to find that my work had fallen short. Instead, to my delight, most of our comments overlapped! Of course, she had points which I had missed completely, but I also had valid questions that had not been brought up in her report.

For once, I felt I had concrete evidence that I wasn’t as incompetent as I often believed – and as early-career researchers are sometimes made to feel. It wasn’t just a well-wisher telling me that I was doing a good job: I could actually see how my input matched the thoughts of someone with much more experience than me.

I went back to work, compiling and writing out a formal report about the manuscript. When sending back the review to the journal, my supervisor let the editor know that I had formally helped: I felt like I was finally part of the scientific community, rather than a mere observer.

I acknowledge that not everybody has an advisor willing to let their graduate students participate in peer review. These days, though, we can take charge of this ourselves by posting reviews of preprints online or participating in initiatives like PREreview. Based on my experience, I hope more of my peers will seize this opportunity.

As an early-career researcher, it’s easy to feel (or be told) that you are not enough, that you don’t have ‘what it takes’, or that your contributions are not as valuable. Yet for me, the journey has only just begun! I still have a long way to go as a scientist — and I still attend talks which everyone seems to get except for me. But on days I doubt my abilities, I think back to my reviewing experience and remember that I should have a little more faith in myself. To me, that makes all the difference.

## Share your experiences

This article is a Sparks of Change column, where people around the world share moments that illustrate how research culture is or should be changing. Have an interesting story to tell? See what we’re looking for and the best ways to get in touch here.

